# Gibberellin and Auxin Influence the Diurnal Transcription Pattern of Photoreceptor Genes via CRY1a in Tomato

**DOI:** 10.1371/journal.pone.0030121

**Published:** 2012-01-17

**Authors:** Paolo Facella, Loretta Daddiego, Giovanni Giuliano, Gaetano Perrotta

**Affiliations:** 1 Italian National Agency for New Technologues, Energy and Sustainable Economic Development (ENA), Trisaia Research Center, Rotondella, Italy; 2 Italian National Agency for New Technologues, Energy and Sustainable Economic Development (ENA), Casaccia Research Center, Rome, Italy; University of Oxford, United Kingdom

## Abstract

**Background:**

Plant photoreceptors, phytochromes and cryptochromes, regulate many aspects of development and growth, such as seed germination, stem elongation, seedling de-etiolation, cotyledon opening, flower induction and circadian rhythms. There are several pieces of evidence of interaction between photoreceptors and phyto-hormones in all of these physiological processes, but little is known about molecular and genetic mechanisms underlying hormone-photoreceptor crosstalk.

**Methodology/Principal Findings:**

In this work, we investigated the molecular effects of exogenous phyto-hormones to photoreceptor gene transcripts of tomato *wt*, as well as transgenic and mutant lines with altered cryptochromes, by monitoring day/night transcript oscillations. GA and auxin alter the diurnal expression level of different photoreceptor genes in tomato, especially in mutants that lack a working form of cryptochrome 1a: in those mutants the expression of some (IAA) or most (GA) photoreceptor genes is down regulated by these hormones.

**Conclusions/Significance:**

Our results highlight the presence of molecular relationships among cryptochrome 1a protein, hormones, and photoreceptors' gene expression in tomato, suggesting that manipulation of cryptochromes could represent a good strategy to understand in greater depth the role of phyto-hormones in the plant photoperceptive mechanism.

## Introduction

During evolution, plants have developed accurate mechanisms to integrate internal signal such as hormones and environmental cues like light and temperature, in order to respond as quickly and efficiently as possible to any change. Several growth and developmental processes, such as seed germination, stem elongation, seedling de-etiolation, cotyledon opening, flower induction and circadian rhythms are activated and/or regulated by both light and hormones, suggesting interactions between signalling pathways [1], [2], [3], [4], [5], [6].

Plants have acquired the tools to monitor precisely the changing intensity and spectrum of light, its direction and, in specific cases, its plane of polarization [7], through a number of photoreceptors: the red (R)/far-red(FR) – absorbing phytochromes and the blue/UV-A – absorbing cryptochromes and phototropins [8], [9].

In *Arabidopsis*, phytochromes are encoded by five different genes, *PHYA* through *PHYE* [10], [11], cryptochromes by three genes, *CRY1*, *CRY2* and *CRY-DASH* [12], [13], [14]. Cryptochromes and phytochromes control several overlapping physiological responses, [15], [16] at all stages of plant development. Although the exact nature of co-action has yet to be well elucidated, it is known that blue light-mediated de-etiolation involves the interaction of both phytochrome and cryptochrome signaling [17], [18], [19].

In tomato (*Solanum lycopersicum*), four cryptochrome genes have been discovered and analyzed so far: two *CRY1*-like (*CRY1a* and *CRY1b*), one *CRY2* and one *CRY-DASH* gene [20], [21], [22]. The role of the *CRY1a* gene has been elucidated through the use of antisense [23] and mutant [24] plants. *CRY1a* controls seedling photomorphogenesis, anthocyanin accumulation, and adult plant development. No effects of *CRY1a* on flowering time or fruit pigmentation have been observed. The overexpression of tomato *CRY2* causes phenotypes similar to but distinct from their *Arabidopsis* counterparts (hypocotyls and internode shortening under both low and high fluence blue light), but also several novel ones, including a high-pigment phenotype, resulting in overproduction of anthocyanins and chlorophyll in leaves and of flavonoids and lycopene in fruits [25]. Tomato *CRY-DASH* gene is under the control of circadian machinery with a light-regulated transcription pattern and it is expressed since the earliest phases of tomato development [22].

In tomato, phytochromes are encoded by five genes: *PHYA*, *PHYB1*, *PHYB2*, *PHYE* and *PHYF* [26]. Phylogenetic analyses showed orthology between *PHYA*, *PHYE* and *PHYC*/*F* gene pairs in *Arabidopsis* and tomato; tomato *PHYB1* and *PHYB2* were originated by an independent duplication [27]. Roles for *PHYA* and *PHYB1* in the mediation of tomato plant de-etiolation responses to red light (R) have been demonstrated previously [28], [29]. Although the *phyAphyB1* double mutant is blind to low-irradiance R, it de-etiolated normally under white light. The phenotype of *phyAphyB1phyB2* mutants under natural daylight indicated an important role for *PHYB2* in this residual response [30] and it also clear that *PHYB2* is also active in R-sensing [31].

Different classes of hormones regulate several aspects of seedling development, often in redundant or antagonistic relationship among them. Gibberellin (GA) and abscisic acid (ABA) are two critical signals with antagonistic effects on seed dormancy and germination [32], [33]. GA and brassinosteroid (BR) are involved in the repression of photomorphogenesis in the dark [34], [35] and with auxin promote hypocotyl elongation [36]. Low levels of auxin induce root growth, whereas high levels have inhibitory effects [37]. Besides, auxin plays an important role in lateral root initiation and growth [38].

The interaction among hormones may be additive, synergistic or antagonistic, making their overall effect more complex (see reviews: [32], [39], [40], [41]). For example, auxin is known to control root growth in part through modulation of the cellular response to GA [42], but it regulates hypocotyl elongation independently of GA [36]. Recent evidence suggests that auxin and BR signaling pathways are overlapping and interdependent: expression of several AUX/IAA genes (SAUR and GH3 homologs) are regulated by both auxin and BR [43], [44], [45].

A few downstream genes are known to modulate or integrate different hormonal signals. For example, the *Arabidopsis sax* mutant provides strong evidence for interaction among multiple hormones related to BR levels [46], [47]. Finally, *SPY* gene was recently demonstrated to have a role as a coordinator in cross-talk between GA and cytokinin [48].

Phyto-hormones also play important roles in regulating vegetative and reproductive development. Mutants with a decreased response to GA, BR or auxin are usually characterized by dwarfism, reduced apical dominance, dark-green foliage, and reduced fertility [32], [49], [50], [51]. GA also regulates flowering time and flower organ development [52], [53].

There are several pieces of evidence of interactions between photoreceptors and hormones during plant development. Many studies have suggested that phytochromes and cryptochromes influence the activities of auxin in order to regulate plant growth. Indeed, PHYA, PHYB and CRY1 promote light-dependent effects of the auxin transport inhibitor 1-N-naphthylphthalamic acid on both hypocotyls and root elongation in *Arabidopsis* [54], [55]. Other reports indicate that cryptochromes regulate the transcription of *AUX/IAA* genes [56] and that *AUX/IAAs* are phosphorylated by PHYA [57].

Gibberellins are known to be a component of light signalling [58]; phytochromes and GA_s_ act in coordination to regulate multiple aspects of *Arabidopsis* development such as flowering and hypocotyls elongation [59], [60], [61]. Phytochromes affect GA levels, by regulating expression of the *GA_2_ox* and *GA_3_ox* genes [62], and may also regulate GA responsiveness [63], [64], [65]. It has been recently shown that PHYA and PHYB mediate light stabilization of the DELLA proteins, which may, at least partially, result from the phytochrome-dependent regulation of GA homeostasis [66].

Light and GA play an antagonistic role during photomorphogenesis [34]. It has been reported that light inhibits the ability of Phytochrome Interacting Factors (PIFs) to promote dark-type growth (elongation of hypocotyl and repression of chloroplast development), through a stabilizing action of PIF proteins in the dark, rather than the destabilization, mediated by activated phytochromes, that occurs in the light. On the other hand, PIF responses are restored by the destabilizing action of GA over DELLA [4], [67].

Phytochromes and GAs are also involved (together with auxins and ethylene) in regulating shade-avoidance responses, that maximize light capture by positioning the leaves out of the shade [68].

In comparison to the phytochrome-regulated responses, the relationship between cryptochromes and GA in the blue light responses is less clear in *Arabidopsis*. It has been found in pea that CRY1 and PHYA redundantly regulate *GA_2_ox* and *GA_3_ox* expression and GA signaling [65], [69]. A recent report demonstrated that cryptochromes mediate blue light regulation of GA catabolic/metabolic genes, which affect GA levels and hypocotyl elongation [5].

Furthermore cytokinins in *Arabidopsis* are involved in the regulation of the circadian clock mechanism [6], in which both cryptochromes and phytochromes are also involved. Besides Vandenbussche and collegues [70] concluded that HY5, a positive regulator of photomorphogenesis induced by CRY1 and CRY2 [71], represents a point of convergence between cryptochrome and cytokinin signalling pathways.

Several other examples of hormone-over-photoreceptor interaction could be reported; however there is little or no information about effects of phyto-hormones on photoreceptors and possible alteration of their gene transcript accumulation.

We decided to investigate the effects of the addition of exogenous phyto-hormones to the photoreceptor system of tomato *wt* and transgenic lines with altered crypthochromes, by monitoring the day/night transcript oscillations. We demonstrated that exogenous GA and auxin are able to modify the tomato photoreceptor diurnal expression patterns, especially in cry1a mutants, suggesting the presence of a molecular network among cryptochrome 1a, hormones, and photoreceptor genes in tomato.

## Results and Discussion

To investigate whether phyto-hormones influence the diurnal expression pattern of the tomato cryptochrome (*CRY1a, CRY1b, CRY2 AND CRY-DASH*) and phytochrome (*PHYA, PHYB1, PHYB2, PHYE AND PHYF*) genes, we have exogenously added citokinin (t-zeatin), gibberellic acid (GA_3_), auxin (IAA) and abscisic acid (ABA) phyto-hormones to *wt* tomato, to a mutant genotype with a non functional CRY1a (*cry1a-*) [24] and to a transgenic line overexpressing the cryptochrome 2 (*CRY2OX*) [25]. All tomato plants were grown hydroponically under a light cycle of 16 h light/8 h darkness (LD), as described in Methods. Two hours before the presumptive dawn (ZT-2) a specific phyto-hormone (t-zeatin, GA_3_, IAA or ABA) was added (for details, see Methods). Aerial components of the hormone-added plants and control plants (without hormone) were sampled at distinct time points over a diurnal cycle (ZT0, ZT6, ZT12, ZT16 and ZT20) and subjected to cryptochrome and phytochrome gene expression assays, by Q-RT PCR. We further analyzed the diurnal transcription pattern of two genes for which the transcription is strictly light-regulated: *GIGANTEA (GI)*, involved in the regulation of the plants' circadian rhythm [72] and *CAB4*, a member of the large family of Chlorophyll a/b-binding proteins [73].

The effects of cryptochrome alterations on the photoreceptors' transcription pattern, without hormone treatment, are relatively minor, with the obvious exception of the fact that *CRY2* transcripts are constantly up-regulated in *CRY2OX* genotype. Furthermore, *GI* and *CAB4* transcripts show the widest day/night oscillation and a sharp peak at 12 h and 6 h after dawn, respectively; the different genotypes influence the peak amplitude rather than the phase of the cycling transcripts (Fig S1). Transcript alteration patterns similar to the above mentioned ones have already been observed in our previous work carried out using soil grown plants in LD [74]. However, in hydroponically grown plants we don't have strong effects on other *CRYs* and *PHYs* transcripts except for significant down-regulation of some photoreceptor transcripts (*CRY1a, CRY1b, CRY-DASH, PHYA, PHYB1, PHYE, PHYF*) at several time points in CRY2 over-expressing tomatoes (Fig. S1).

**Figure 1:**
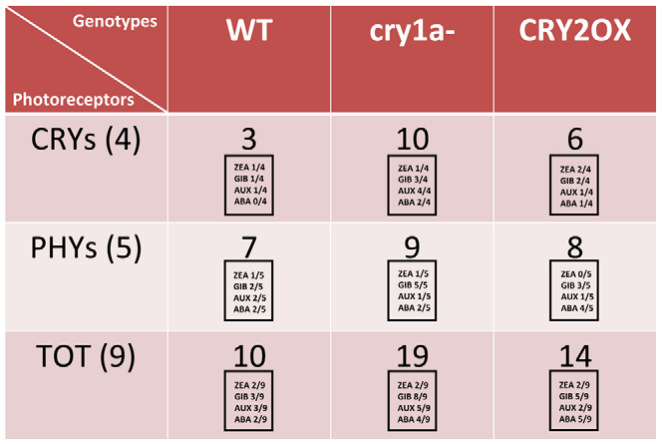
Number of transcription patterns altered in at least three points per cycle, by ZEA, GIB, AUX and ABA phyto-hormones in *wt*, *cry1a-* and *CRY2OX* genotypes. We considered four cryptochrome (CRYs (4)) and five phytochrome (PHYs (5)) gene transcripts. In the squares is indicated the number of altered patterns for each hormone.

**Figure 2:**
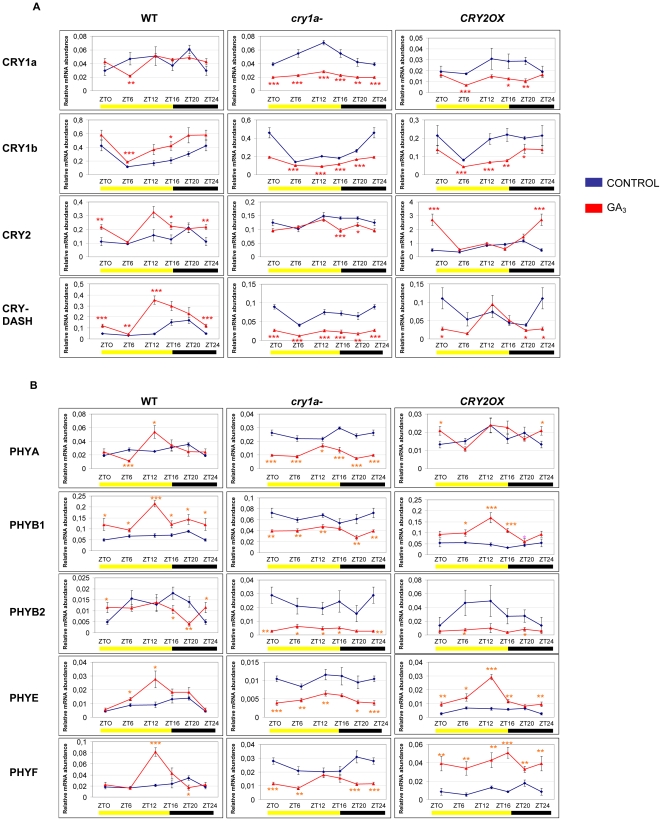
Diurnal expression pattern of Cryptochrome (A) and Phytochrome (B) transcripts analyzed by QRT-PCR in *wt*, *cry1a*- and *CRY2OX* GA_3_-treated tomato plants. Results are presented as a ratio after normalization with β-actin. Yellow and dark bars along the horizontal axis represent light and dark periods, respectively. Time points are measured in hours from dawn (zeitgeber Time [ZT]); data at ZT24 constitute a replotting of those at ZT0. The control data, of gene expression in the absence of hormone applications, are reproduced, for clarity, from those in Figure S1. Data shown are the average of two biological replicates, with error bars representing SEM. Hormone-treated plant transcripts significantly different from the corresponding ones of control plants are marked with a * (Student's t test, P≤0.05), two ** (Student's t test, P≤0.01) and three *** (Student's t test, P≤0.001).

**Figure 3:**
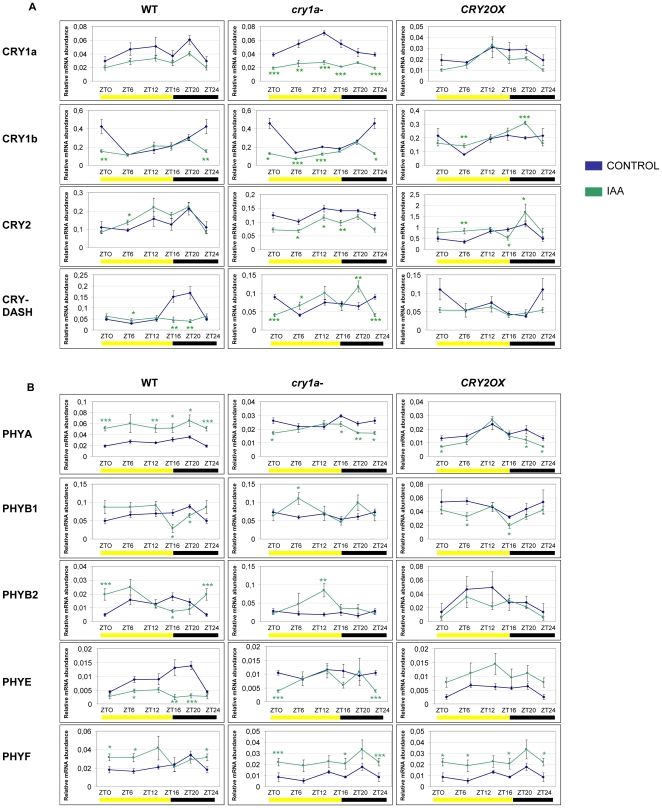
Diurnal expression pattern of Cryptochrome (A) and Phytochrome (B) transcripts analyzed by QRT-PCR in *wt*, *cry1a*- and *CRY2OX* IAA-treated tomato plants. Results are presented as a ratio after normalization with β-actin. Yellow and dark bars along the horizontal axis represent light and dark periods, respectively. Time points are measured in hours from dawn (zeitgeber Time [ZT]); data at ZT24 constitute a replotting of those at ZT0. The control data, of gene expression in the absence of hormone applications, are reproduced, for clarity, from those in Figure S1. Data shown are the average of two biological replicates, with error bars representing SEM. Hormone-treated plant transcripts significantly different from the corresponding ones of control plants are marked with a * (Student's t test, P≤0.05), two ** (Student's t test, P≤0.01) and three *** (Student's t test, P≤0.001). Data from control plants are replotted from Figure 2.

**Figure 4:**
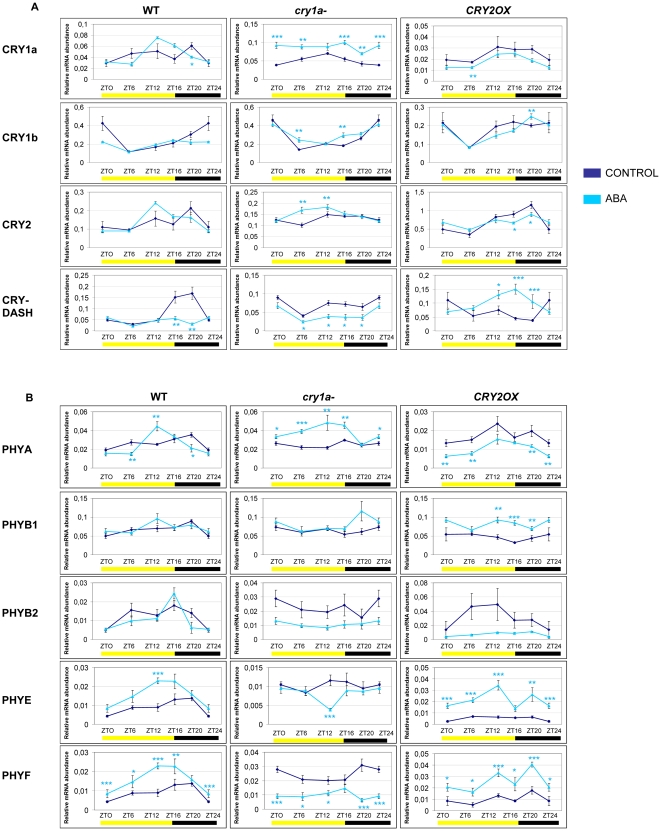
Diurnal expression pattern of Cryptochrome (A) and Phytochrome (B) transcripts analyzed by QRT-PCR in *wt*, *cry1a*- and *CRY2OX* ABA-treated tomato plants. Results are presented as a ratio after normalization with β-actin. Yellow and dark bars along the horizontal axis represent light and dark periods, respectively. Time points are measured in hours from dawn (zeitgeber Time [ZT]); data at ZT24 constitute a replotting of those at ZT0. The control data, of gene expression in the absence of hormone applications, are reproduced, for clarity, from those in Figure S1. Data shown are the average of two biological replicates, with error bars representing SEM. Hormone-treated plant transcripts significantly different from the corresponding ones of control plants are marked with a * (Student's t test, P≤0.05), two ** (Student's t test, P≤0.01) and three *** (Student's t test, P≤0.001). Data from control plants are replotted from Figure 2.

**Figure 5:**
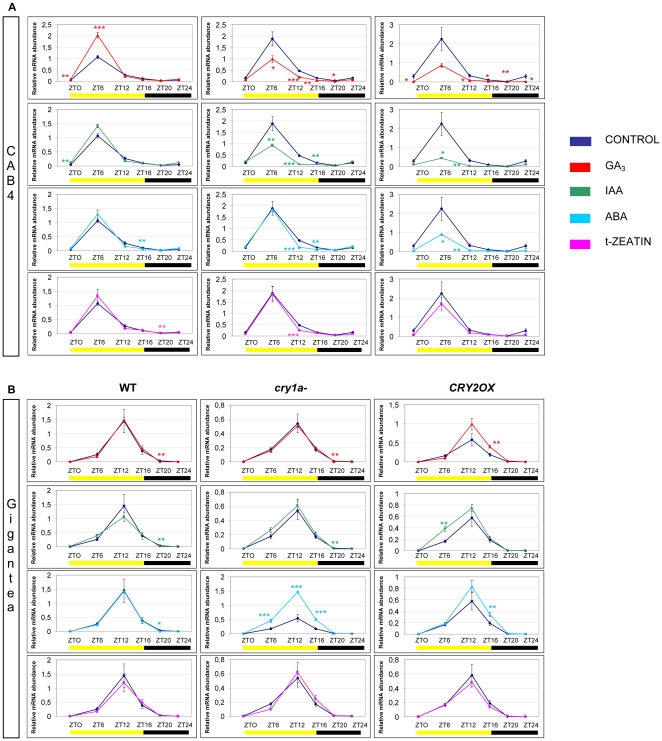
Diurnal expression pattern of *CAB4* (A) and *GIGANTEA* (B) transcripts analyzed by QRT-PCR in *wt*, *cry1a*- and *CRY2OX* hormone-treated tomato plants. Results are presented as a ratio after normalization with β-actin. Yellow and dark bars along the horizontal axis represent light and dark periods, respectively. Time points are measured in hours from dawn (zeitgeber Time [ZT]); data at ZT24 constitute a replotting of those at ZT0. The control data, of gene expression in the absence of hormone applications, are reproduced, for clarity, from those in Figure S1. Data shown are the average of two biological replicates, with error bars representing SEM. Hormone-treated plant transcripts significantly different from the corresponding ones of control plants are marked with a * (Student's t test, P≤0.05), two ** (Student's t test, P≤0.01) and three *** (Student's t test, P≤0.001).

The reciprocal interaction between light and phyto-hormones is a well-known physiological process: light was found to regulate directly the biosynthesis of active gibberellins [75], ethylene [76] and ABA, as well [58]. The molecular mechanisms that regulate this interaction during plant development and life remain unclear, although they are starting to be unraveled [77]; here we provide evidence of a remarkable level of control of gibberellin and auxin on cryptochrome and phytochrome gene expression in tomato. Our results show that this control varies according to the analyzed genotype (Fig. 1). In general, the genotype with non functional cryptochrome 1a, *cry1a-*, appears to be much more sensitive to exogenous hormones than *wt* (Fig. 1). The data regarding CRY2 expression in *CRY2OX* genotype showed that the presence of an overexpression construct driven by a constitutive promoter is presumably able to dilute any hormonal effects on the transcription of this cryptochrome (Fig. 2A, 3A, 4A, 5A).

### Effects of phyto-hormones on tomato photoreceptor diurnal transcription

The modification of cryptochrome and phytochrome transcription pattern following addition of GA_3_ is remarkable, especially in *cry1a*- plants (Fig. 1 and Fig. 2AB). In this genotype, GA_3_ produces strong downregulation of both cryptochrome and phytochrome transcripts, with the only exception of *CRY2*, at all time points (Fig. 2A). The lack of a functional CRY1a protein produces a generic and strong signal of downregulation of the photo-perceptive apparatus of tomato in GA_3_ treated plants with regard to the untreated ones, suggesting a pivotal role for CRY1a in mediating light and gibberellin stimuli. Analyzing in greater detail the behavior of cryptochrome transcripts following GA_3_ treatment in *wt* tomato plants, it is evident that cryptochromes are quite unaffected by rapid change of hormone concentration in the culture medium, the only exception being the upregulation of *CRY-DASH* (Fig. 2A). On the other hand, in *CRY2OX* and *cry1a-* genotypes cryptochrome 1 transcripts are mostly downregulated (Fig. 2A). This hints that CRY1a and CRY2 play an antagonistic role in *CRY1a* and *CRY1b* transcriptional regulation, when gibberellin is added.

The transcription pattern of the phytochrome gene family following treatment with GA_3_, evidenced an opposite response in *cry1a*- plants with respect to *wt* and *CRY2OX* tomatoes (Fig. 2B). Indeed, when a functional form of CRY1a protein is absent, all five phytochromes are constantly downregulated (Fig. 2B); on the contrary, when CRY1a works normally (in *wt* and *CRY2OX* plants) the same genes, but *PHYB2*, appear to be mostly upregulated, especially at ZT12 (Fig. 2B). These results demonstrate that the presence of a CRY1a working protein is a decisive factor for transcript regulation of phytochrome genes. This effect is particularly evident in *PHYB1* transcription (Fig. 2B), suggesting a possible role of *PHYB1* in regulating the molecular network among hormones, photoreceptors and light in tomato, as an element downstream of CRY1a.

The photoreceptor response to auxin (IAA) treatment is lower than to that of gibberellin (Fig. 3AB). Once again, the most sensitive genotype to exogenous hormone is clearly *cry1a-*, especially when focusing on the cryptochrome mRNA transcripts: *CRY1a*, *CRY1b* and *CRY2* are downregulated in at least three time points analyzed (Fig. 3A). In *wt* and *CRY2OX* plants no clear pattern of up or downregulation of cryptochrome transcripts was observed (Fig. 3A). CRY1a may play a crucial role in the regulation of chryptochrome expression also under auxin stimulus; however, this role seems to be absent for phytochromes, which are almost totally unaffected in *cry1a-* plants (Fig. 3B). Therefore, the action of CRY1a over tomato photoreceptor gene transcripts changes according to different hormonal stimuli. Within the phytochrome family only PHYA appears to be sensitive to auxin treatment: indeed, in *wt* plants this gene is up regulated across the day; this effect is not visible in *cry1a-* and *CRY2OX* plants (Fig. 3B), suggesting that CRY1a and CRY2 can play a positive and a negative role, respectively, in the auxin induced alteration of *PHYA* transcripts.

Generally, ABA does not cause dramatic effects on transcription of cryptochrome genes. Nevertheless some very interesting exceptions must be remarked: the strong upregulation of *CRY1a* and the downregulation of *CRY-DASH* in *cry1a-* plants, as well as, the upregulation of *CRY-DASH* in *CRY2OX* tomatoes (Fig. 4A). It is interesting to note that the transcription of *CRY-DASH*, whose function as photoreceptor has been heavily discussed [78], [79], is influenced by the other two main tomato cryptochromes, at least under hormonal stimulus.

Analyzing phytochrome responses to exogenous ABA in *CRY2OX* treated-plants, we observed strong upregulation during the day for *PHYB1*, *PHYE* and *PHYF*; conversely, *PHYA* shows downregulation (Fig. 4B).

In ABA treated *cry1a-* plants the scenario is completely inverted: *PHYA* is upregulated at all time points, but ZT20 (presumptive night); contrarily, *PHYF* is constantly downregulated, with the sole exception of ZT16 (Fig. 4B). In *CRY2OX* genotype *PHYA* appears to be downregulated with the exception of ZT12 and ZT16, whereas *PHYE* and *PHYF* are up regulated during almost all cycle (Fig. 4B). In general, cryptochrome 1–2 type proteins seem to play a role in phytochrome responses to ABA treatment, in accordance with what was already discussed for gibberellin treatment.

The effect of exogenous t-zeatin on photoreceptor gene expression is very weak and quite independent from the genotype (Fig. S2 and Fig. 1).

### Effects of phyto-hormones on tomato *CAB4* and *GIGANTEA* diurnal transcription

Transcription of the photosynthetic gene *CAB4* is unaffected by the addition of exogenous t-zeatin in all three genotypes under study (Fig. 5A). On the contrary, GA_3_-treatment generates significant upregulation in *wt* plants and a downregulation in both *cry1a*- and *CRY2OX* genotypes, especially during the light phase of the day (Fig. 5A). It is surprising that in *wt* genotype gibberellin can stimulate the expression of a gene like *CAB4*, implicated in the perception of light stimuli, when *Arabidopsis spy* mutant, that is hypersensitive to GA, presents a pale phenotype, very similar to photoreceptors mutants [80]. Our data suggest that the upregulation of *CAB4* is probably driven by CRY1a and antagonized by CRY2, since they are downregulated in both mutant and overexpressor genotypes after GA_3_-treatment (Fig. 5A).

A similar situation is evident in auxin-treated plants except that the addition of IAA does not interfere with *CAB4* transcription in *wt* genotype, providing evidence that addition of auxin can alter *CAB4* transcription only as a consequence of the abnormal presence of functional cryptochromes (Fig. 5A). Furthermore, downregulation of *CAB4* is also evident after ABA treatment but limited to *CRY2OX* plants (Fig. 5A), evidencing a specific dose-effect of the cryptochrome 2 over ABA induced transcript alterations.

It is known that the expression of the circadian and flowering gene *GI* is (at least partially) under the control of cryptochromes in *Arabidopsis* [81], and, more specifically, under the control of CRY1a in tomato [74]. Our results here reveal that *GI* transcripts are not affected by exogenous adding of t-zeatin, gibberellin and auxin in all the three genotypes observed (Fig. 5B); on the contrary, *GI* is very sensitive to ABA, but only in *cry1a*- plants, where its transcripts are dramatically upregulated during the part of the day in which *GI* is more expressed (from ZT6 to ZT16) (Fig. 5B). In a recent work [74], we have already demonstrated that the lack of an active form of CRY1a causes downregulation of *GI*; these new experiments highlight that in *cry1a-* plants CRY1a and ABA signaling components are redundant in maintaining optimal *GI* expression, resulting, most likely, in fine modulation of numerous important physiological processes in tomatoes.

### Concluding remarks

The main finding of this work is that without a functional cryptochrome 1a, both GA_3_ and IAA can perturb the diurnal expression pattern of tomato photoreceptors: GA_3_ downregulates both cryptochrome and phytochrome expression pattern, whereas IAA is able to downregulate cryptochrome diurnal transcription.

Data presented here reveal a substantial degree of control of cryptochromes (especially CRY1a) over the regulatory networks formed by phytohormones, light and photoreceptors. We demonstrated that cryptochromes have a main role in the regulation of the diurnal expression pattern of both cryptochrome and phytochrome genes under hormonal stimulus. Particularly, the absence of a working CRY1a protein makes “the tomato system” more sensitive to changes of phyto-hormone concentration in the growing medium.

In *cry1a-* tomatoes, most photoreceptors, especially phytochromes, become repressible by GA addition. The loss of photoperception via CRY1a is able to compound the skotomorphogenic phenotype caused by gibberellin action, as in that combined situation the transcription of most other photoreceptors is also repressed; CRY2 overexpression can, in some cases (*PHYB1*, *PHYE*),antagonize this action.

Moreover, under the given treatments, cryptochrome 1a, and in a milder manner cryptochrome 2, can regulate not only the expression of photoreceptor gene transcripts, but also the transcription pattern of genes involved in photosynthetic processes and circadian rhythm, as *CAB4* and *GI*. This hints a major involvement of phyto-hormones in mediating the physiological response of plants to light stimuli by an interaction with photoreceptors.

## Materials ands Methods

Standard molecular biology protocols were followed as described in Sambrook and colleagues [82].

### Plant material

All experiments were carried out in *Solanum lycopersicum* (cv Moneymaker) background, *cry1a-* (80B mutant) and transgenic *CRY2OX* seeds [24], [25]. Tomato seeds were germinated in standard paper towels. After germination, uniform seedlings were placed into transparent plastic boxes (14 seedlings of the same genotype per box) and grown hydroponically for 28 days in a growth chamber in LD conditions (16 h light/8 h dark-25°C) without humidity control. Light intensity of about 50 µmol m^−2^ s^−1^ was provided by Osram (Munich) 11–860 daylight lamps. The composition of the full nutrient solution used during the plant growth was: 1 mM MgSO_4_, 2.5 mM Ca(NO_3_)_2_, 2 mM KNO_3_, 0.1 mM K_2_HPO_4_, 10 µM Fe-EDDHA, 10 µM B, 2 µM Mn, 1 µM Zn, 0.5 µM Cu, 0.2 µM Mo, 0.2 µM Co, 0.2 µM Ni and 25 µM Cl [83]. Nutrient solution was replaced in each box every 2 days. The solution pH was maintained at 7.5 with CaCO_3_. At ZT -2 (ZT- Zeitgeber time = number of hours after the onset of illumination) [84] of the 29^th^ day of growth, 20 µM phyto-hormones were added to nutrient solution of test-plants (this hormone concentration is within experimental ranges commonly used pharmacologically for a given phyto-hormone, and is within a physiologic range); control-plants were let in the standard nutrient solution. The aerial parts of 10–14 plants for each genotype (*wt*, *cry1a-* and *CRY2OX*) both for treated and control plants were harvested at the times shown.

### Quantitative RT-PCR

Total RNA (1 µg) was reverse-transcribed with oligo-dT and Superscript III (Invitrogen), according to the manufacturer's instructions. First strand cDNA (5 ng) was used as template for QRT-PCR. QRT-PCR assays were carried out with gene-specific primers, using an ABI PRISM 7900HT (Applied Biosystems) and the Platinum SYBR Green master mix (Invitrogen), according to manufacturer's instructions. The primer sequences are:

CRY1a TCCTTGCTAACTTTTTGTTAGTATCTGTG; TACGATCTTTTGTTAGCCTGCCT

CRY1b: ATATCGATGTAATGCAAGAACTATGGA; TCTGGTACAGAGAAGTAGAGGCATCA

CRY2: CAAAGGGTGCCATCAATGC; GCTTGTTATCATTGAGCTTCTTTGTT

CRY-DASH: GACACTCTCCTGGAATGATG; CACCAGTCTTCTTGGTATATCC

PHYA: GAATCGAAGGTGACTATAGAGCGATT; GAACACCAGCCAAATTGATCAG

PHYB1: GGGCTTCCTCCTGAATTGG; GCTCAGTCCTAGGCCTTCCTG

PHYB2: TGATTTCTTACAGATTATGGCAAGCT; TTGGTCGAAGATGGACTTCTACC

PHYE: TTGCTTAGTGTAGTGCACCATGC; GTTTCAAACCAGGTAACACCTTGA

PHYF: TTGAGCAAGGATCAAAGGCA; GTGTCGTCAATGATCTTGGCTAGT

GI: GCAACCATTGGAAAACAAAG; CAGACAGAAGCAAGGACATAAG

CAB: GAAGGGTCCAATTGAGAAC; GTACAAAGTTTGTCCCGTAAG

ACTIN: AGGTATTGTGTTGGACTCTGGTGAT; ACGGAGAATGGCATGTGGAA.

PCR conditions were: 5 min at 95°C, followed by 45 cycles at 95°C for 15 sec, and at 58°C for 60 sec. At the end of the PCR, the thermocycler has been programmed to generate a thermal denaturation curve of the amplified DNA and to measure the melting temperature of the PCR product(s). The shape of the melting curve indicates whether the amplified products are homogeneous and the melting temperature provides confirmation that the correct product has been specifically amplified. Relative template abundance was quantified using the relative standard curve method described in the ABI PRISM 7900HT manual and the data were normalized for the quantity of the β-actin transcript [85], an housekeeping gene whose transcripts do not oscillate during the day (data not shown). A serial dilution of 10-, 100-, 1000-,10000-, and 100000-fold of each studied gene fragment was used to determine the amplification efficiency of each target and housekeeping gene. At least three PCR runs were carried out for each cDNA to serve as technical replicates and two independent experiments were carried out by using two biological replicates for each genotype. Means from two independent experiments were subjected to SEM calculation, Student's t test using PAST.

## Supporting Information

Figure S1 **Effect of CRY1a loss-of-function and CRY2 over-expression on diurnal expression of tomato cryptochrome (A), phytochrome (B) and** ***GIGANTEA*** **/** ***CAB4*** **(C) genes.** *Wt*, *cry1a*- and *CRY2OX* tomato plants were grown hydroponically under LD conditions. The abundance of the mRNAs was measured by QRT-PCR. Results are presented as a proportion of the highest value after normalization with β-actin. Yellow-black box along the horizontal axis represents light and dark periods, respectively. Time points are measured in hours from dawn (zeitgeber Time [ZT]); data at ZT24 constitute a replotting of those at ZT0. Data shown are the average of two biological replicates, with error bars representing SEM. Time points of *CRY2OX* and *cry1a-* genotypes, significantly different from the corresponding ones in *wt* genotype are marked with a * (Student's t test, P≤0.05), two ** (Student's t test, P≤0.01) and three *** (Student's t test, P≤0.001).
(DOCX)

Figure S2 **Diurnal expression pattern of Cryptochrome (A) and Phytochrome (B) transcripts analyzed by QRT-PCR in** ***wt*** **,** ***cry1a*** **- and** ***CRY2OX*** **t-ZEATIN-treated tomato plants.** Results are presented as a ratio after normalization with β-actin. Yellow and dark bars along the horizontal axis represent light and dark periods, respectively. Time points are measured in hours from dawn (zeitgeber Time [ZT]); data at ZT24 constitute a replotting of those at ZT0. The control data, of gene expression in the absence of hormone applications, are reproduced, for clarity, from those in Figure S1. Data shown are the average of two biological replicates, with error bars representing SEM. Hormone-treated plant transcripts significantly different from the corresponding ones of control plants are marked with a * (Student's t test, P≤0.05), two ** (Student's t test, P≤0.01) and three *** (Student's t test, P≤0.001). Data from control plants are replotted from Figure 2.
(DOC)
