# Supplementary material for: Gibberellin and Auxin Influence the Diurnal Transcription Pattern of Photoreceptor Genes via CRY1a in Tomato
Source: PLoS One. 2012 Jan 17;7(1):e30121. doi: 10.1371/journal.pone.0030121 (PMC3260215; doi:10.1371/journal.pone.0030121)
Supplement: Figure S1 — Effect of CRY1a loss-of-function and CRY2 over-expression on diurnal expression of tomato cryptochrome (A), phytochrome (B) and GIGANTEA / CAB4 (C) genes. Wt, cry1a- and CRY2OX tomato plants were grown hydroponically under LD conditions. The abundance of the mRNAs was measured by QRT-PCR. Results are presented as a proportion of the highest value after normalization with β-actin. Yellow-black box along the horizontal axis represents light and dark periods, respectively. Time points are measured in hours from dawn (zeitgeber Time [ZT]); data at ZT24 constitute a replotting of those at ZT0. Data shown are the average of two biological replicates, with error bars representing SEM. Time points of CRY2OX and cry1a- genotypes, significantly different from the corresponding ones in wt genotype are marked with a * (Student's t test, P≤0.05), two ** (Student's t test, P≤0.01) and three *** (Student's t test, P≤0.001). (DOCX) [file pone.0030121.s001.docx]

Figure 1S

**A**

**WT**

***cry1a-***

***CRY2OX***

**B**

**C**
