# Supplementary material for: Gibberellin and Auxin Influence the Diurnal Transcription Pattern of Photoreceptor Genes via CRY1a in Tomato
Source: PLoS One. 2012 Jan 17;7(1):e30121. doi: 10.1371/journal.pone.0030121 (PMC3260215; doi:10.1371/journal.pone.0030121)
Supplement: Figure S2 — Diurnal expression pattern of Cryptochrome (A) and Phytochrome (B) transcripts analyzed by QRT-PCR in wt , cry1a - and CRY2OX t-ZEATIN-treated tomato plants. Results are presented as a ratio after normalization with β-actin. Yellow and dark bars along the horizontal axis represent light and dark periods, respectively. Time points are measured in hours from dawn (zeitgeber Time [ZT]); data at ZT24 constitute a replotting of those at ZT0. The control data, of gene expression in the absence of hormone applications, are reproduced, for clarity, from those in Figure S1. Data shown are the average of two biological replicates, with error bars representing SEM. Hormone-treated plant transcripts significantly different from the corresponding ones of control plants are marked with a * (Student's t test, P≤0.05), two ** (Student's t test, P≤0.01) and three *** (Student's t test, P≤0.001). Data from control plants are replotted from Figure 2. (DOC) [file pone.0030121.s002.doc]

Figure 2S

CONTROL

t-ZEATIN

**CRY1a**

**CRY1b**

**CRY2**

**CRY-DASH**

**WT *cry1a- CRY2OX***

**A**

CONTROL

t-ZEATIN

**PHYA**

**PHYB1**

**PHYB2**

**PHYE**

**PHYF**

**WT *cry1a- CRY2OX***

**B**
